# A Minimal Metric for the Characterization of Acoustic Noise Emitted by Underwater Vehicles

**DOI:** 10.3390/s20226644

**Published:** 2020-11-20

**Authors:** Giacomo Picardi, Clara Borrelli, Augusto Sarti, Giovanni Chimienti, Marcello Calisti

**Affiliations:** 1 The BioRobotics Institute, Scuola Superiore Sant’Anna, 56127 Pisa, Italy; marcello.calisti@santannapisa.it; 2 Department of Excellence in Robotics & AI, Scuola Superiore Sant’Anna, 56127 Pisa, Italy; 3 Dipartimento di Elettronica, Informazione e Bioingegneria, Politecnico di Milano, 20133 Milano, Italy; clara.borrelli@polimi.it (C.B.); augusto.sarti@polimi.it (A.S.); 4 Department of Biology and CoNISMa LRU, University of Bari Aldo Moro, 70125 Bari, Italy; giovanni.chimienti@uniba.it

**Keywords:** underwater robotics, acoustic noise, underwater noise, ROV, underwater legged robots, ego-noise, visual census, benthic environment

## Abstract

Underwater robots emit sound during operations which can deteriorate the quality of acoustic data recorded by on-board sensors or disturb marine fauna during in vivo observations. Notwithstanding this, there have only been a few attempts at characterizing the acoustic emissions of underwater robots in the literature, and the datasheets of commercially available devices do not report information on this topic. This work has a twofold goal. First, we identified a setup consisting of a camera directly mounted on the robot structure to acquire the acoustic data and two indicators (i.e., spectral roll-off point and noise introduced to the environment) to provide a simple and intuitive characterization of the acoustic emissions of underwater robots carrying out specific maneuvers in specific environments. Second, we performed the proposed analysis on three underwater robots belonging to the classes of remotely operated vehicles and underwater legged robots. Our results showed how the legged device produced a clearly different signature compared to remotely operated vehicles which can be an advantage in operations that require low acoustic disturbance. Finally, we argue that the proposed indicators, obtained through a standardized procedure, may be a useful addition to datasheets of existing underwater robots.

## 1. Introduction

The oceans are essential for life on Earth, hosting a remarkable and still poorly known biodiversity, representing an invaluable source of food and energy, and being involved in the balancing of the planet’s climate [1,2,3]. Thanks to the advances in marine robotics, the last decades represented a turning point for marine operations worldwide, from scientific explorations to commercial activities. The development of underwater vehicles, such as Remotely Operated Vehicles (ROVs), submersibles, and Autonomous Underwater Vehicles (AUVs) has allowed a large range of operations that were previously impossible using scuba diving techniques due to the fact of both time and depth limitations.

Depending on the specific task required, different types of underwater vehicles can be currently used. For instance, AUVs are often used for long operations such as monitoring ecological parameters or mapping the seabed over wide areas. Missions are usually pre-programmed with little or no interactions with human operators except for the deployment and recovery phases. Instead, ROVs are used in missions that require the continuous supervision of an external operator, including maintenance interventions on anthropogenic structures, search-and-recovery missions and scientific surveys. All underwater robots are equipped with different sets of sensor-packs, which can be as simple as a single camera used as a feedback for the operator of small commercial ROVs, up to a whole sensory suite including navigation units, sonar-based equipment, and sophisticated devices for ecological and biological investigations in AUVs and larger ROVs.

Although appearing silent, the oceans are characterized by a wide spectrum of sounds. Some of them are natural, such as the noise produced by the waves or emitted by animals [4], and some are anthropogenic, generated by human activities such as shipping, recreational boating or industrial activities, and generally identified as “noise pollution”. Underwater vehicles and robots also produce acoustic noise when they are operated in the sea, and this aspect must be faced by users for a two-fold motivation. On one side, anthropogenic underwater noise can have a negative effect on marine species and, on the other, the noise generated by the robots may affect the quality of the collected data, for example, by interfering with the acoustic sensors in the lower frequency band (<5 kHz) [5,6,7] in case of remote sensing studies or disturbing marine fauna in case of biological surveys [8,9,10,11].

In terrestrial robotics, the noise produced by a device represents an issue that has recently gained attention under the name of ego-noise [12]. Essentially, the noise produced by a robot moving is recorded by microphones along with useful signals and may deteriorate the performance of sound-based algorithms such as speech recognition or acoustic-based navigation. While for the terrestrial case different techniques have been proposed to estimate and filter out the ego-noise [12,13], there are only a few works addressing this problem for underwater robotics [7].

The ego-noise of aquatic robots is an essential variable to take into account in studies that target the assessment of vagile fauna presence and composition, considering that it can disturb the organisms, thus limiting the chances to see them. For instance, ROVs can be used to highlight the pivotal role of important habitats, such as coral forests, in enhancing the presence of species of conservation interest as well as of fish and crustaceans of high commercial value. For instance, the forests of the bamboo coral *Isidella elongata* (Esper, 1788) [14] proved to be an essential habitat for a rich species assemblage, including species of fish and crustaceans of high commercial value that thrive on soft bottoms [11]. A comparable role was observed in other coral forests such as, among others, those structured by the pink sea fan *Eunicella verrucosa* (Pallas, 1766) [15] or by the black coral *Antipathella subpinnata* (Ellis and Solander 1786) [16] in the Mediterranean Sea, as well as monospecific or mixed-coral assemblages all over the Atlantic Ocean [15,16,17]. However, these studies are certainly biased by the noise produced by the ROV that scares the animals with the risk of underestimating their presence and distribution. Besides large mammals and sea turtles, which have been widely known for being very sensitive to noise disturbances (e.g., [18]), fish and marine invertebrates can also be affected by underwater sounds [19]. For example, crustaceans can detect, produce, and respond to sound [11], while fish can change their foraging behavior or show other signs of stress under anthropogenic noise disturbances [9,10,20]. Propellers are major sources of acoustic noise for ROVs and, as already observed at larger scale for boats [8,21], their noise can significantly scare the fish fauna. For this reason, studies aiming to assess the vagile fauna composition often benefit from the use of landers (i.e., fixed observation platforms equipped with light, camera, and eventually bait [22]) with clear limitations on the deployment and movement of the tool during the survey.

In the literature, there are a few attempts at characterizing the noise generated by an underwater robot, most of them with the goal of achieving stealth in military applications [23,24]. Regardless of the reason for carrying out such a characterization, all works have used a setup involving hydrophones (attached to the robot, externally positioned or both) and a controlled recording environment, such as an empty swimming pool or ad hoc facilities, to minimize acoustic reflections. The complexity of the measurement, together with the recent awareness for this specific issue, are probably among the reasons why the datasheets of commercially available and research underwater robots do not report indicators of their noise signature. However, as the employment of underwater robots in the observation and monitoring of marine fauna increases, the demand for rapid and general methods to evaluate the noise of different underwater robots will grow as well.

In this work, we resorted to cameras directly mounted on three different underwater robots with the aim of providing a simple characterization of the noise they introduce to the environment for different maneuvers in terms of well-known signal processing tools. The methodological choice of using commercial cameras instead of specific sensors, such as hydrophones, was driven by two main motivations. On the one hand, recordings occurred during real operative sessions, i.e., the robots were performing their task in the field and were not operated specifically for the collection of acoustic data. On the other hand, such a simple setup would allow end-users to perform the same analysis and understand how much noise they are introducing into the environment where they are operating. The consequences of this methodological choice are discussed in Section 4 of this work. We performed our analysis on the recordings obtained from two robots belonging to the ROV class, which, at the time of writing, was the only commercially available option for remotely operated explorations by means of robotic devices. We compared the results with a recently introduced Underwater Legged Robot (ULR) [25] that represents a complementary approach to underwater operations [26,27]. Based on the analysis performed and accounting for the simplicity of the recording setup, we proposed two indicators to provide an intuitive and easily obtainable description of the noise signature of underwater robots, i.e., the spectral roll-off point, $f_{RO}$ [28], and the noise introduced to the environment, NE. Results revealed that the ULR robot class presented both a lower $f_{RO}$ and NE compared to the tested ROVs, suggesting that this novel category may represent a valuable alternative to existing underwater robots for operations that require a low noise signature.

## 2. Materials and Methods

The robots used in this study fall into two very different classes. On one hand, the ROVs, Prometeo and Seaeye Falcon DR (Falcon), are slightly positively buoyant for automatic resurfacing in the case of failure and propel by means of thrusters. On the other hand, the ULR SILVER2, which is negatively buoyant and harnesses the interaction between legs and seabed to move. While there are several commercially available ROVs, ULRs are a novel category of devices mostly used in a research context at the time of writing. An overview of the features of the robots used in this work is presented in Table 1.

Prometeo is propelled by four thrusters actuated by brushless DC motors. The propellers are functionally divided into elevators and thrusters, respectively, responsible for vertical and horizontal motion. In this work Prometeo was operated in manual mode (i.e., the operator had direct control of each propeller) because it is not equipped with autopilot. This allowed to clearly distinguish phases during which only the elevators or the thrusters were active, phases in which all propellers were active, and phases when all motors were off.

Falcon is propelled by five thrusters actuated by brushless DC motors. The thrusters’ configuration is different from the Prometeo, as it presents one vertical thruster responsible for vertical motion plus four horizontal thrusters. In this work the Falcon was operated with the autopilot on, so that it was not possible to isolate sequences when specific thrusters were on or off, but it recorded the effective noise produced by the analyzed maneuvers. This choice was driven by the fact that autopilot is commonly used by pilots to stabilize the vertical position of the ROV, thus measuring the noise produced with autopilot on gave a more reliable idea about the effective noise produced during operations at sea.

The SILVER2 is characterized by six legs 63 cm long, actuated by 18 Dynamixel servomotors controlled using user-tunable feed-forward strategies. As thoroughly described in Reference [25], SILVER2 is capable of hopping on the seabed by means of periodic extensions and retractions of the springy legs and of walking with each leg following a predefined path.

**Figure 1:**
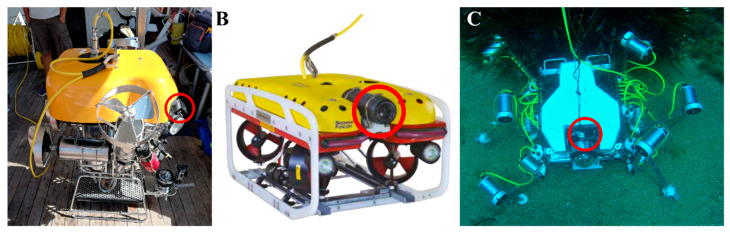
Underwater robots used in this work: (**A**) Prometeo, (**B**) Falcon, and (**C**) SILVER2. The cameras used for recording are marked with a red circle.

Audio signals were recorded with cameras directly mounted on the structure of the robots as shown in Figure 1. For Prometeo and SILVER2, a GoPro enclosed in the proprietary waterproof case was attached to the structure of the robot. For the Falcon, the audio signals were recorded by the onboard camera (Kongsberg HD color zoom camera provided by the producer) mounted on the upper-front side of the robot. Unavoidably, the distance between the microphones of the cameras and the actuators, which represent the main source of noise, was different from robot to robot due to the fact of their configurations and their operability. In particular, in the case of Prometeo, the elevators and the thrusters were, respectively, 40 cm and 90 cm away from the GoPro. For the Falcon, the front horizontal thrusters, rear horizontal thrusters, and vertical thruster were respectively 85 cm, 30 cm, and 55 cm away from the camera. In the case of SILVER2, the distance between the actuators and the camera was not constant because the legs were mobile, and it goes from a minimum of 35 cm for the front legs to a maximum of 95 cm for the rear legs; however, the movement is periodic and limited by legs workspace, so this should not affect the presented analysis.

Recordings occurred in independent sessions, during real operations of the devices in different areas of the Italian coast (Mediterranean Sea), all of them with good weather conditions and with supporting vessel engines off. Data from Prometeo were recorded at Tremiti Islands Marine Protected Area (Italy, Adriatic Sea) during a scientific study on vulnerable coral forests at a 55–60 m depth [15]. Data from Falcon were recorded at the Aeolian Islands (Italy, Tyrrhenian Sea) during marine explorations of the seabed [29] at 55–60 m depth. Data from SILVER2 were recorded in two different sessions in a shallow stretch of water in Livorno (Italy, Tyrrhenian Sea) at a depth of 0.8 m. Each robot performed different maneuvers as described in Table 2 and shown in the Supplementary Materials Video S1. For all sessions, a recording of the ambient noise was obtained when all actuators were off. The raw data are available as Supplementary Materials.

Each audio signal *x*(*t*) was obtained by trimming a long mp3 file of the duration of the whole session to extract only the part corresponding to the maneuver of interest. The sampling frequency was *f_s_* = 44,100 Hz, and it was assumed that x(t)=s(t)+n(t), where *s*(*t*) is the sound emitted by the robot and *n*(*t*) is the ambient noise recorded when all actuators are off. The following analysis was performed:

1. Power spectral density computed with a Hanning window of length *N*, over *N* bins, where *N* is the total number of samples of *x*(*t*);
2. Spectral roll-off point $f_{RO}=i$ such that $\sum_{k=b_{1}}^{i}s_{k}=\alpha \sum_{b_{1}}^{b_{2}}s_{k}$, where *b*_1_ and *b*_2_ are the band edges, *s_k_* is the spectral value at bin k and α∈(0,1). The spectral roll-off point represents the frequency before which α percent of the total energy of the signal lies. In this work α=0.95, (*b*_1_, *b*_2_) = (0, 10^4^);
3. Spectrogram computed on 1024 sample bins with a 768 sample overlap;
4. Autocorrelation coefficient $r=\frac{1}{\left|r\left(0\right)\right|}E\left\{x_{n+m}x_{n}^{*}\right\}$, where the operator E{} is the expected value operator, and the normalization makes r(0)=1.
5. Noise introduced into the environment $NE=10\log_{10}\frac{E\left[x\left(t\right)\right]}{E\left[n\left(t\right)\right]}$.

## 3. Results

### 3.1. Raw Signals

**Figure 2:**
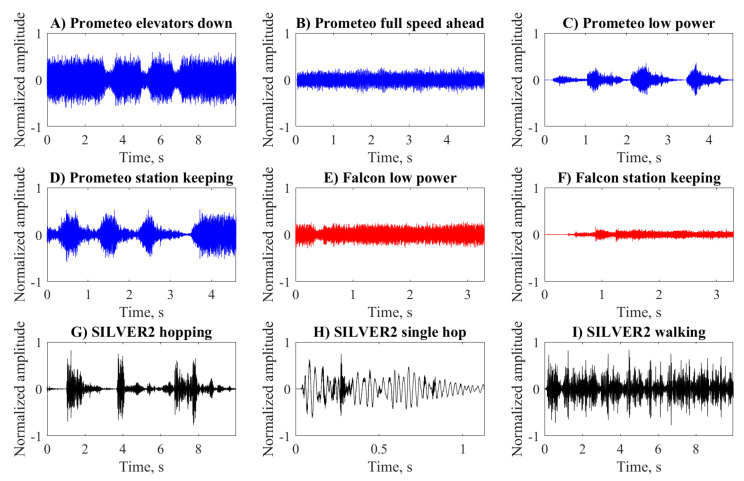
Raw mp3 signals of Prometeo (blue), Falcon (red), and SILVER2 (black) performing various maneuvers.

The raw mp3 signals for the three robots performing the maneuvers described in Table 2 are reported in Figure 2. The resulting acoustic signals from the Prometeo ROV (Figure 2A–D) were characterized by a wide spectrum, and the differences among them were marked by lower intensity regions associated to the moments when the operator released the throttle as in the low power and station keeping maneuvers. The higher intensities were recorded in the elevators down and station keeping maneuvers, obtained by activating the elevators in order to maintain the ROV on the seabed. This intense use of the elevators, which are closer to the camera (Figure 1), supports the higher intensity observed in these maneuvers with respect to the full speed ahead (Figure 2).

In the case of the Falcon ROV (Figure 2E,F), the raw signals present trended very similar to the Prometeo, with higher frequency dynamics than SILVER2. Although the intensities were lower compared to the first ROV, they cannot be directly compared since the thrusters were positioned differently on the two robots and different cameras with different cases were used.

The SILVER2 was operated in two distinct modes, resulting in different acoustic signatures (Figure 2G–I). The hopping signal presented high-intensity bursts linked with the periodic activation of motors, followed by silent phases during which the robot passively glided in the water. On the other hand, the sound emitted during walking was more uniform over time because in this mode, the legs continuously followed predefined paths without pauses.

**Figure 3:**
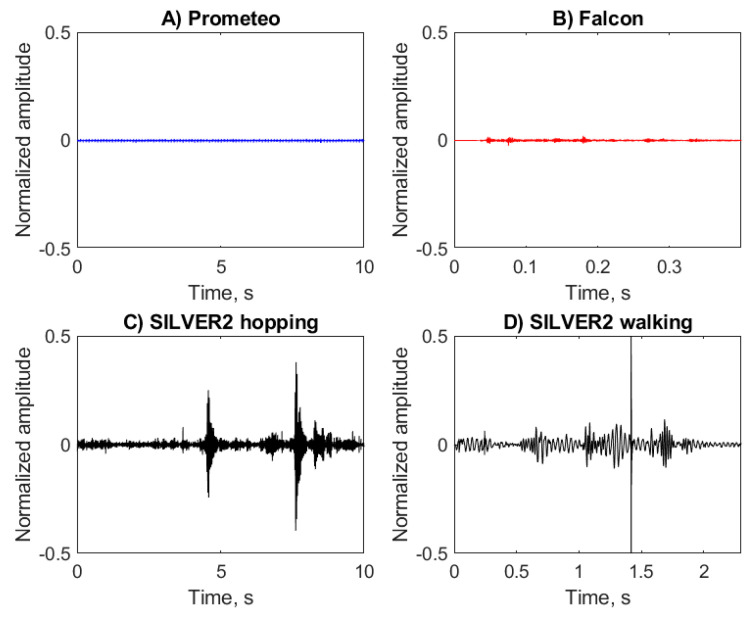
Raw mp3 signals of ambient noise for the four recording sessions considered.

The signals presented in Figure 2 were recorded in four different sessions (one each for Prometeo and Falcon and two for SILVER2) characterized by differences in the location and depth. For each session a recording of the ambient noise (AN) was obtained when all actuators were off (Figure 3). The ANs of Prometeo (Figure 3A) and Falcon (Figure 3B) were very low because they were acquired at a depth of around 60 m in marine areas with limited human presence. On the other hand, the recording sessions for SILVER2 (Figure 3C,D) took place in a shallow body of water (approximately 0.8 m) so that the acquired ANs presented higher intensities and localized bursts, probably connected with the effects of wind, small waves or human activities.

### 3.2. Spectra and Spectrograms

**Figure 4:**
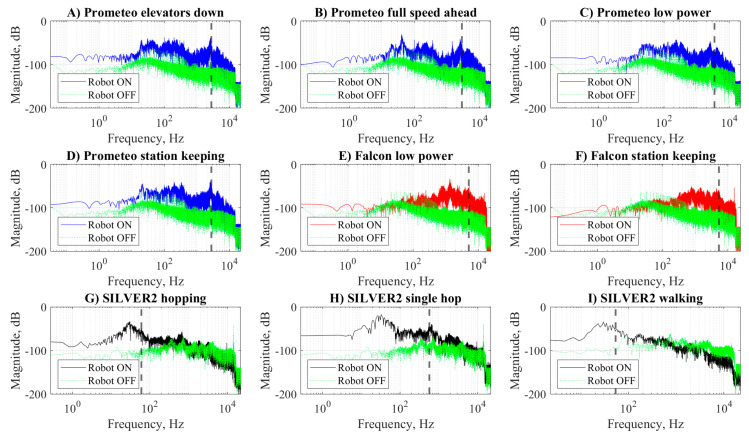
Power spectral density of Prometeo (blue), Falcon (red), and SILVER2 (black) performing various maneuvers. For each maneuver, the power spectral density of the ambient noise recorded when the robots were off is reported (green). The vertical dotted line indicates the spectral roll off point of each $f_{RO}$ of each signal. The reference value for the magnitude in dB was $1/\sqrt{2}$.

The power spectral density plots reported in Figure 4 confirmed some of the observations on the raw signals presented in the previous section. Indeed, the ROV class robots (Prometeo and Falcon) presented a wider bandwidth compared with the ULR SILVER2. In particular, the spectra of Prometeo (Figure 4A–D) were characterized by three consecutive peaks in the range 0.03–2.5 kHz. The spectra of Falcon (Figure 4E,F), instead, were characterized by a single peak at around 1.3 kHz and they had no lower frequency peaks. No marked differences of the spectra related to the different maneuvers were observed for both the ROVs. The spectra of SILVER2 (Figure 4G–I) were all characterized by a peak at around 0.03 kHz followed by a rapid decrease. The differences in the energy distribution along the spectra were also reflected by the spectral roll-off point $f_{RO}$, here computed for α=0.95 and marked by a vertical dashed line in Figure 4, that has values in the range of 2.7–5.3 kHz for Prometeo, 2.9–3.5 kHz for the Falcon, and only 0.05–0.6 kHz for SILVER2. In the case of a single hop from SILVER2, instead of a longer sequence, less energy was found at lower frequency and consequently $f_{RO}$ reached 0.6 kHz.

The power spectral densities of the ANs of the four recording sessions are reported in Figure 4. In the case of Prometeo and Falcon, they were spread and presented maxima lower than −50 dB. On the other hand, the AN of SILVER2 presented a low-frequency peak in correspondence of the peaks observed in Figure 4.

**Figure 5:**
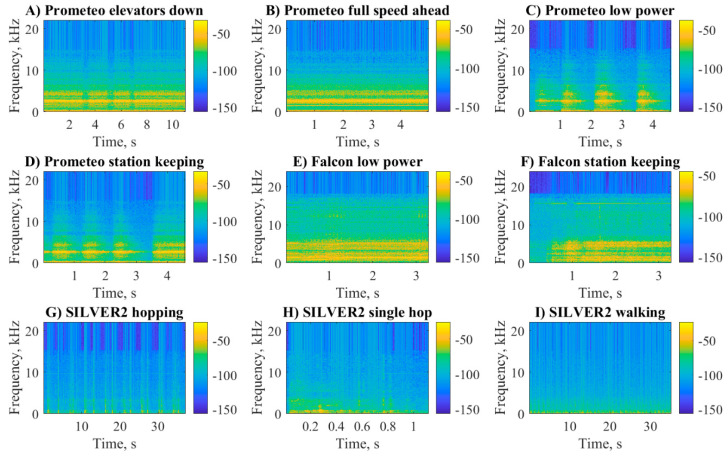
Spectrograms of Prometeo (blue), Falcon (red), and SILVER2 (black) performing various maneuvers. The magnitude of the spectra is color-coded according the color bars to the right of each plot.

The spectrograms of all the maneuvers considered in this work are shown in Figure 5. The higher frequency content of the acoustic signals produced by the ROVs (Figure 5A–F) with respect to the ULR (Figure 5G–I) is clear, as well as the intermittent nature of some maneuvers such as the low power and station keeping of Prometeo or the hopping of SILVER2. In the case of Prometeo (Figure 5A–D), we can notice a bright yellow horizontal line corresponding to around 2.6 kHz and two less intense bands in the ranges 4−5 kHz and 0–0.5 kHz. In the case of Falcon (Figure 5E,F), instead, we notice two bands in the ranges 0.7–2.7 kHz and 3.5–5 kHz. Such bands were not present in the spectrograms related to SILVER2 (Figure 5G,I) for which the highest power reached was in the lowest frequencies of the spectrum (<0.2 kHz). In a similar analysis [7], such horizontal bands were related to the revolution per minute (RPM) of the propellers; however, this information is not available for the presented experiments.

### 3.3. Autocorrelation

**Figure 6:**
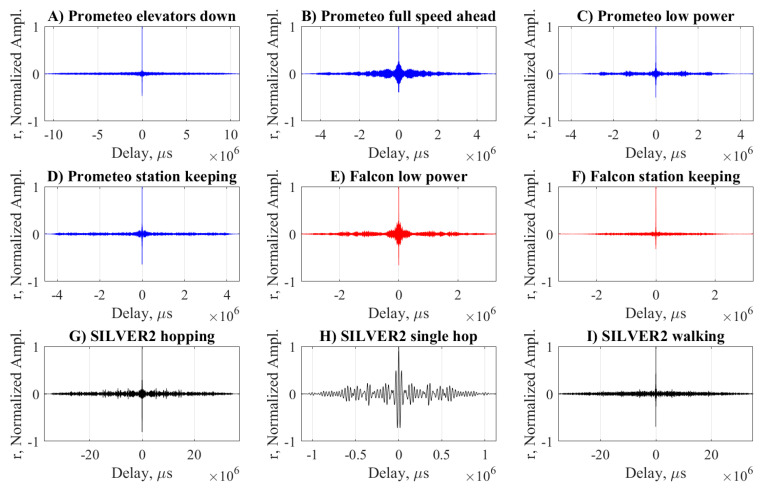
Autocorrelation coefficient *r* of the recordings of Prometeo (blue), Falcon (red), and SILVER2 (red) performing various maneuvers. Notice the different scale of the *x*-axis, required to keep a high resolution.

An intuition of the periodicity of the acoustic signature emitted by the robots clearly emerges by the autocorrelation coefficients depicted in Figure 6. Here the ROV class robots (Figure 6A–F) produced very tight plots, meaning non-periodic signals as opposed to SILVER2 (Figure 6G–I). Not surprisingly, when only a single hop is considered, the periodicity which characterizes the hopping maneuver is lost.

### 3.4. Noise Introduced into the Environment

**Figure 7:**
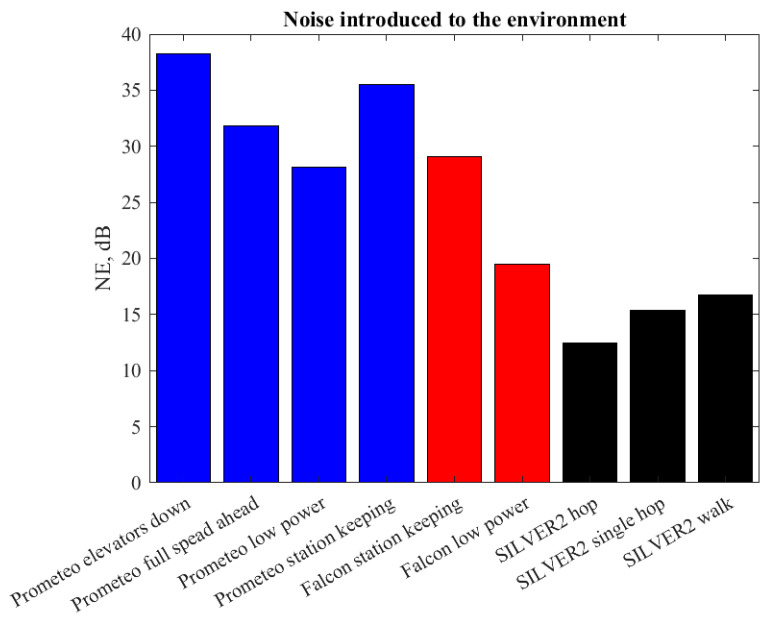
Noise introduced to the environment by Prometeo (blue), Falcon (red), and SILVER2 (black) performing different maneuvers.

As already commented, the absolute acoustic intensities recorded in this work cannot be compared directly because the orientation and distance of the microphone from the actuators can vary from case to case and according to the recording device. For this reason, in order to provide a ground of comparison for the noise emitted by the robots considered, we computed the NE indicator for all the maneuvers and reported it in Figure 7. As expected from experience in the deployment of these vehicles, the ROVs broadly introduced a higher level of noise in the environment with respect to the ULR SILVER2.

## 4. Discussion

All robots moving produce a noise that is mostly related to the type, power, and activation pattern of its actuators, and which can be an obstacle to a successful completion of some specific tasks. The ego-noise may limit the ability of a humanoid or service robot to perform speech recognition to assist humans [12] or it may complicate audition-based navigation [30].

Given the very low transmission of electromagnetic waves, underwater systems often rely on sonar technologies for sensing. Although the operating frequencies of such devices are generally in the order of MHz, in some cases, when a longer range is needed [5,6], the ego-noise spectra of underwater robots (Figure 4) may overlap with the ones of the sensing devices. Furthermore, when underwater robots are used for observation of marine fauna, the ego-noise certainly overlaps with the hearing frequencies of most fish, which is generally below 5 kHz [31].

In this work we approached the issue of ego-noise from the perspective of underwater robotics and characterized the acoustic noise emitted by three different robots belonging to the categories of ROV and ULR in terms of power spectral density, spectral roll-off point, spectrogram, autocorrelation and noise emitted to the environment. Similar analyses have been performed in literature, mostly with the aim of achieving stealth in military applications or improve the signal-to-noise ratio in autonomous surveys [7]. In this work, we acquired the data with a very simple setup consisting of a camera mounted directly on the vehicles (Figure 1) and recording during real operating conditions. Data obtained in this way were not suitable for a sophisticated analysis, based on critical working assumption, nevertheless they allow to easily provide a simple yet intuitive characterization of the noise emitted by underwater robots and perform a first order classification based on acoustic noise emitted which is lacking at the time of writing and may be of great help for operators of underwater robots who want to get an idea of the acoustic noise that they are introducing into the environment where they are experimenting.

Comparisons among robots based on the absolute intensity of the raw signals or power spectral densities measured in this study would be misleading. In fact, the noise produced by the three robots tested was not recorded in the same location and with the same device, which had distances from the actuators (which are considered the main source of noise) that were not constant. On the other hand, it is possible to understand in which frequency range most of the energy of the signal is concentrated by either looking at the power spectral density plots reported in Figure 4 or by resorting to the synthetic indicator, $f_{RO}$, which represents the frequency behind which 95% of the energy of the signal lies. Furthermore, we introduced the NE indicator computed as a signal-to-noise ratio in which the signal was the acoustic signal recorded for any maneuver *x* and the noise was the AN recorded when all motors were off *n*. The NE allows to compare the noise emitted in the environment and recorded with the presented setup in non-controlled conditions by explicitly *subtracting* the energy related to the ambient noise. Among all the maneuvers considered, the most interesting result concerns the NE of station keeping. For the Prometeo and Falcon ROVs, station keeping consisted of compensating, either manually or through a feedback control, the buoyancy with continuous or intermittent use of vertical thrusters. The SILVER2 instead realizes station keeping by relying on negative buoyancy and just keeping all motors off. For this reason, the recording of SILVER2 during the station keeping maneuvers was the same as the AN and therefore NE = 0.

These evaluations are of critical importance for the use of ROVs and/or ULRs in scientific surveys aiming to observe vagile organisms such as fish, mollusks, and crustaceans, that can be easily disturbed by the ego-noise of the robot. In fact, marine fauna is particularly sensitive to noise pollution, including the one eventually introduced during the surveys [9,10,11], de facto biasing the data collected in terms of species presence and abundance. These surveys represent a valid example of the need of silent vehicles, with the double purpose of avoiding acoustic stress on the fauna and collect reliable data about the animal community. In many cases, these surveys are still carried out with highly destructive methods, such as trawling or dredging, in order to collect the animal species. On the contrary, non-invasive techniques based on visual surveys are needed, particularly when surveying sensitive habitats of great ecological importance such as the so-called vulnerable marine ecosystems [32,33].

The possibility to develop a silent tool able to explore the seabed and to perform long station keeping on specific targets would allow a non-invasive assessment of vagile macro- and megafauna, as well as the observation of unknown behaviors, leading to the understanding of species’ roles and interactions in remote environments, such as the mesophotic and aphotic portions of the seabed [34]. Currently, ROVs are the only commercially available option for teleoperated underwater robotic operations. In addition, ULRs, among which SILVER2 is one of the most recent representatives, are capable of moving directly onto the seabed and coupling very good passive station keeping performance with relatively agile mobility [25]. Such features make ULR complementary to ROVs and a viable option to solve the trade-off often faced by marine biologists between a highly mobile, yet often noisy ROV and a very silent yet still lander platform [34]. The intuition about low noise signature of ULRs anticipated in Reference [25] was confirmed by the indicators proposed in this work with SILVER2 presenting the lowest NE and $f_{RO}$ among the robots tested.

To conclude, in this work the proposed indicators (i.e., NE and $f_{RO}$) have been obtained with the simple presented setup. Such simplicity may allow end-users to derive them for their current mission and, thus, be aware of the noise introduced during operations. On the other hand, it makes them subject to variability which especially depends on environmental conditions and sensing devices. A further methodological weakness of the proposed setup relates with the limited sampling rate, relatively poor sensitivity of the sensing device used and the use of the compressed mp3 standard. For this reason, the analysis could only be performed up to 20 kHz and with low reliability in the low frequency range (0−100 Hz). This may hide potential overlaps between the ego-noise of the underwater robot and the most used sonar technologies, but on the other hand, it allows to evaluate if the ego-noise represents a disturbance in the hearing frequency range of most fish, which is usually below 5 kHz [31].

As future works, the performed measurements can be repeated in a standardized recording setup [35]. The sea ambient noise and the underwater robot noise should be acquired separately using the same exact configuration. Regarding the choice of the acquisition device, an omnidirectional hydrophone is preferred over a standard microphone. Given the goal of our study, the hydrophone should have a frequency response able to reach at least 5 kHz and a sensitivity that allows to capture both low amplitude signals (for ambient noise recording) and medium amplitude signals (for underwater robot noise). When measuring the noise introduced by the underwater robot, the position of the hydrophone should be fixed and in far-field with respect to the position of the robot, i.e., in a position far enough from the underwater robot such that it can be approximated as an acoustic point source. In order not to record acoustic reflections from the surface nor the effects of the waves, the hydrophone should be positioned closer to the seabed. The hydrophone should be connected to an amplifier and to an analog–digital converter. An in situ calibration of the recording system should be performed. Regarding the digital conversion step, sampling frequency and bit depth should reflect the frequency and dynamic range of the analyzed acoustic events, such as for the hydrophone choice. Finally, a lossless format should be preferred, e.g., WAV.

Within this acquisition setup, the proposed indicators may extend their utility and be a valuable addition to datasheet of commercially available underwater vehicles and guide end-users in the selection of the most suitable device for their needs.

## 5. Conclusions

In this work we proposed a simple setup to acquire acoustic data emitted by underwater robots performing different maneuvers with the aim of easily characterize their acoustic signature. We found that the spectral roll-off point, $f_{RO}$, and the noise introduced to the environment NE were effective descriptors that provided an intuitive characterization of the acoustic noise produced by an underwater vehicle operating in a specific environment. Such metrics, obtained with the presented setup are easy to calculate, but prone to variability. By resorting to a standardized protocol which indicates the characteristics of the sensing device and of the ambient noise, they would provide a more general description of the acoustic signature of underwater robots and may be included in the datasheet of commercially available underwater robots to help in the selection of the device most suitable for the requirements of end-users. We performed the proposed analysis on two ROVs and the ULR SILVER2, finding that the latter presented significantly lower NE and $f_{RO}$ for all the maneuvers considered. This result further qualifies ULR has a viable alternative to traditional underwater robots for benthic operations that require a low acoustic disturbance.

## Figures and Tables

**Table 1:** Overview of relevant information of the robots used in this work.

	Prometeo	Falcon	SILVER2
**Category**	ROV	ROV	ULR
**Dimensions (cm)**	80 × 90 × 95	100 × 50 × 60	40 × 55 × 63
**Dry weight (kg)**	55	60	22
**Actuation**	4 brushless DC motors	5 brushless DC motors	18 servomotors
**Power**	1.8 kW VAC	2.8 kW VAC	0.1 kW DC
**Control system**	Manual	Autopilot	Feedforward legged locomotion strategies

ROV = remotely operated vehicles; AUV = autonomous underwater vehicles; VAC = voltage in alternating current.

**Table 2:** All the maneuvers performed by the robots in this study.

Robot	Maneuver	Description
Prometeo	Elevators down	Descend with only elevators active
	Full speed ahead	Forward propulsion at maximum thrust
	Low power maneuver	Coordinated maneuver at low power
	Station keeping	Active buoyancy compensation with elevators
Seaeye Falcon DR	Low power maneuver	Coordinated maneuver at low power
	Station keeping	Feedback control of vertical thruster to stabilize depth
SILVER2	Hopping	Periodic impulsive extension and retraction of legs
	Single hop	One period of hopping
	Walking	Predefined path following of each leg

## Supplementary Materials

The data analyzed and presented in this work are available online at https://www.mdpi.com/1424-8220/20/22/6644/s1.
